# Associations of physical activity volume and intensity with depression symptoms among US adults

**DOI:** 10.3389/fpubh.2025.1592961

**Published:** 2025-04-30

**Authors:** Jikai You, Jing Long, Zezhong Wang, Yanan Yang

**Affiliations:** ^1^School of Physical Education, Jiangxi Normal University, Nanchang, China; ^2^Department of Health Management, Faculty of Military Health Service, Naval Medical University, Shanghai, China

**Keywords:** accelerometer, depression symptoms, physical activity, MIMS, adults

## Abstract

**Background:**

This study aimed to investigate the associations of physical activity (PA) volume and intensity with the risk of depression symptoms.

**Methods:**

The data utilized in this study came from the 2011–2014 National Health and Nutrition Examination Survey. PA was measured using a triaxial accelerometer and calculated using the Monitor-Independent Movement Summary (MIMS), MIMS units are a novel metric derived from wrist-worn accelerometer data, representing the intensity of PA for each minute across the entire monitoring period. PA volume and intensity were expressed by the average of daily accumulated MIMS (Daily MIMS) and peak 30-min MIMS (Peak-30_MIMS_; Peak 30-min intensity), respectively. Depression symptoms were defined as Patient Health Questionnaire-9 score ≥10. Weighted logistic regression and restricted cubic splines were used to evaluate the associations between PA metrics and depression symptoms.

**Results:**

After adjusting for all covariates, higher Daily MIMS and Peak-30_MIMS_ were associated with lower depression risk. Each additional 1,000 units in Daily MIMS and 1-unit in Peak-30_MIMS_ were associated with a 5% [Odds ratio (OR) = 0.95, 95% confidence interval (95% CI): 0.94, 0.98] and 2% (OR = 0.98, 95% CI: 0.97, 0.99) reduction in depression risk, respectively. When including both MIMS metrics in the same model, the association between Peak-30_MIMS_ and depression remained significant (*p* = 0.02), whereas Daily MIMS did not (*p* = 0.60). The spline analysis indicated a monotonic decrease in the OR with higher Daily MIMS values (*P* for non-linear = 0.21). An initial increase followed by a decrease in OR was observed with rising Peak-30_MIMS_ values (*P* for non-linear <0.01).

**Conclusion:**

Our findings indicate that higher PA volume and intensity are associated with lower depression risk. The association between PA volume and reduced depression risk was negated after adjusting for PA intensity in US adults.

## 1 Introduction

Depression is a major public health issue, affecting more than 300 million people worldwide, with increasing prevalence rates each year (1). The World Health Organization predicts that major depression will be the leading cause of the global burden of disease by 2030 (2, 3). Identifying factors that contribute to depression is essential for developing effective public health prevention strategies and delivering targeted health communication.

Many studies have indicated that physical activity (PA) have protective effects against depression symptoms (4, 5). A meta-analysis encompassing eight randomized controlled trials has provided consistent evidence that PA can reduce depression across children, adults, and older adults (6). However, the majority of previous studies on PA have relied on the Global Physical Activity Questionnaire (GPAQ) (7, 8). Despite their utility, self-reported interview data have some limitations, including the variability in respondents’ perceptions of activity intensity and the difficulty in recalling and quantifying physical activity periods (9). In contrast, accelerometers are advanced wearable devices capable of objectively measuring the intensity and duration of an individual’s PA in daily life over prolonged periods (10, 11). A synthesized meta-analysis of eight studies indicated that in the general population, the maximum reduction in all-cause mortality risk associated with accelerometer-measured moderate to vigorous physical activity (MVPA), about 60%, was approximately twice the magnitude found in studies using self-reported PA (12). The use of an accelerometer could facilitate the revelation of the true extent of the correlation between PA and depressive symptoms, thereby improving the personalization of depression treatment strategies.

The National Health and Nutrition Examination Survey (NHANES) in 2020 released physical activity monitor data obtained through wrist accelerometers during the 2011–2014 survey period. John et al. (13) utilized a novel algorithm for monitor-independent movement summary (MIMS) to process data from worn wrist triaxial accelerometers. A key advantage of the MIMS metric lies in its generation through a device-agnostic, universal algorithm, which facilitates cross-study and cross-design comparisons (13). PA volume and intensity were expressed as daily accumulated MIMS (Daily MIMS) (14) and peak 30-min MIMS (Peak-30_MIMS_) (15–18), respectively. Zheng et al. (19) found that higher peak effort and daily accumulation were associated with improved cognitive function. However, to date, few studies have explored the associations of PA volume and intensity with depression symptoms using Daily MIMS and Peak-30_MIMS_.

To address these knowledge gaps, the present study aimed to investigate the associations of PA volume and intensity, as assessed by Daily MIMS and Peak-30_MIMS_, with the risk of depression symptoms among a nationally representative sample of adults, utilizing data from the 2011–2014 NHANES.

## 2 Materials and methods

### 2.1 Study population and design

NHANES is an ongoing, nationally representative, cross-sectional survey of the nutrition and health of the deinstitutionalized US civilian population. It received approval from the National Center for Health Statistics Ethics, all participants provided written informed consent.

**Figure 1:**
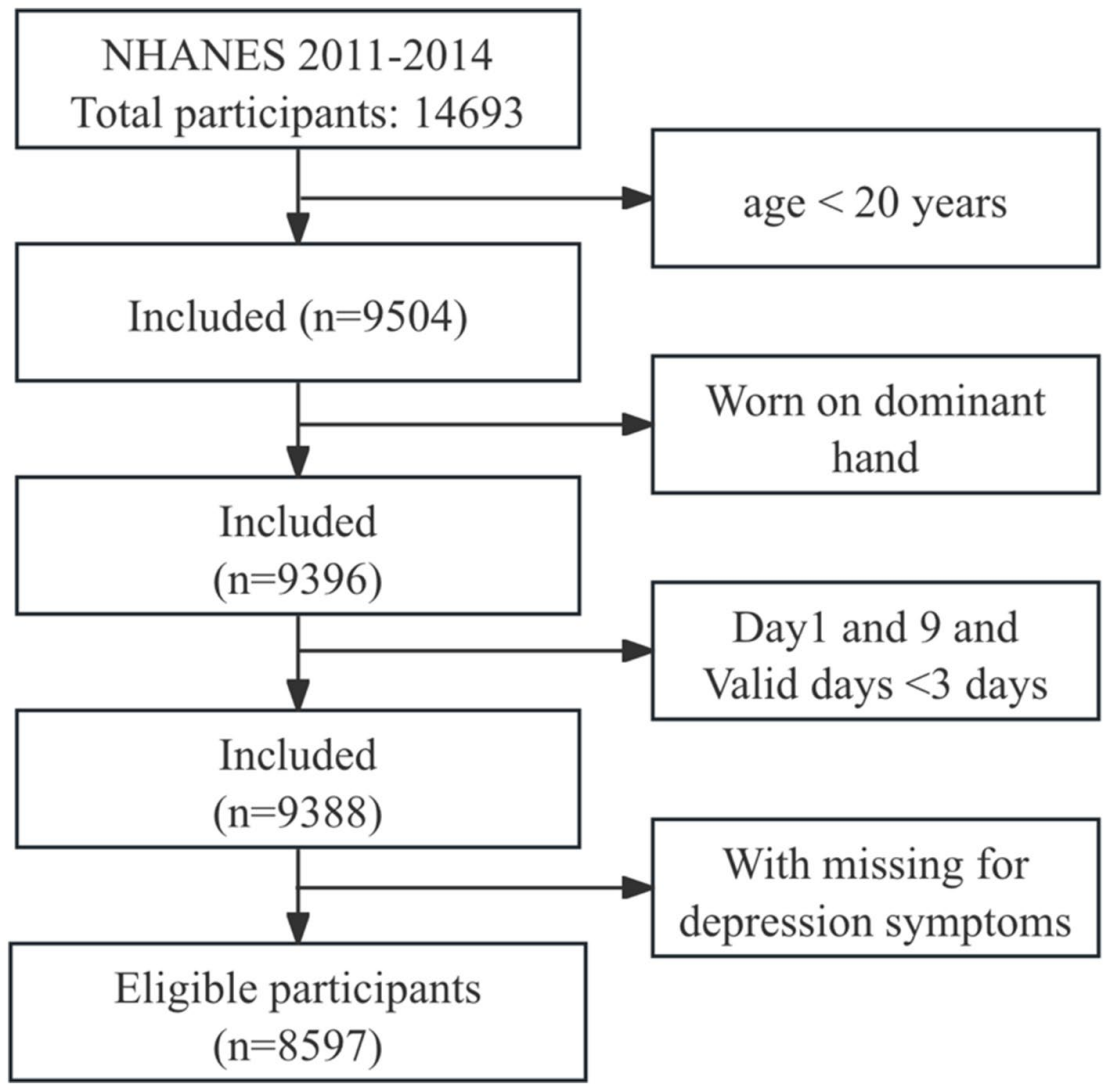
Flowchart of the participants selection.

A total of 14,693 participants with accelerometry data were collected from NHANES 2011–2014. In this study, we included adults aged ≥20 years with ≥3 days of valid accelerometry data, where a valid day was defined as >80% wear time (20, 21). Participants whose accelerometer was worn on the dominant hand and had missing depression symptoms data were excluded. Additionally, data from day 1 and day 9 were excluded due to incomplete 24-h records. Ultimately, 8,597 participants were included in the analysis, with the participant selection process detailed in Figure 1.

### 2.2 Accelerometer data

PA was measured using a triaxial accelerometer (ActiGraph GT3X+, ActiGraph Corp., Pensacola, FL; 80 Hz sampling frequency) worn on the non-dominant wrist for 7 consecutive days. NHANES processes, labels, and summarizes accelerometer data on a minute-by-minute basis, using the MIMS metric. MIMS is a non-proprietary, open-source, device-independent generic summary metric. It shows higher consistency across different devices than other summary metrics (13). MIMS/min data was obtained from the NHANES released in 2022, and device wear status was determined by an open-source machine learning algorithm that identified for each minute device wear status (22). Daily MIMS was calculated by summing the MIMS values for the day, and then mean number of valid days was noted. Peak-30_MIMS_ was calculated by identifying the highest 30 MIMS/min values, averaging these 30 values, and then averaging the result over the valid days.

### 2.3 Depression symptoms

In this research, depression symptoms were identified by a Patient Health Questionnaire-9 (PHQ-9) score of ≥10 (23, 24). This cut-off point is widely applied in depression research, has a validated sensitivity and specificity of 88% (25).

### 2.4 Covariates

We selected these covariates, referencing related previous studies (26–28), including demographic factors (sex, race/ethnicity, education, and age), body mass index (BMI; obesity ≥30 kg/m^2^, overweight: 25–29.9 kg/m^2^, normal weight: 18.5–24.9 kg/m^2^, low weight: <18.5 kg/m^2^) and hypertension. Hypertension was defined by meeting at least one of the following criteria: systolic blood pressure ≥140 mmHg, diastolic blood pressure ≥90 mmHg, or a diagnosis by a healthcare professional (27).

### 2.5 Statistical analysis

The data for analysis were weighted with the requirements of the National Center for Health Statistics using “*survey*” package in R. Participants were classified into two groups based on their depression symptomatology. Custom R scripts was used to compute Daily MIMS (MIMS/day), Peak-30_MIMS_ (MIMS/min), and to calculate the weighted quartiles of MIMS metrics. In addition, smooth curve was used to fit the relationship between age and MIMS metrics. Continuous variables were expressed as means ± standard deviation, and categorical variables were represented by frequency (weighted percentage).

Weighted multiple logistic regression and restricted cubic spline (RCS) with three knots were used to estimate the relationship between each MIMS metric (Daily MIMS, Peak-30_MIMS_) and depression symptoms, adjusting for covariates. In addition, we constructed a model that includes both Daily MIMS and Peak-30_MIMS_ to investigate depression symptoms. All data were analyzed using R 4.3.1 statistical software, and *p* < 0.05 was considered statistically significant.

## 3 Results

### 3.1 General characteristics of included individuals

**Figure 2:**
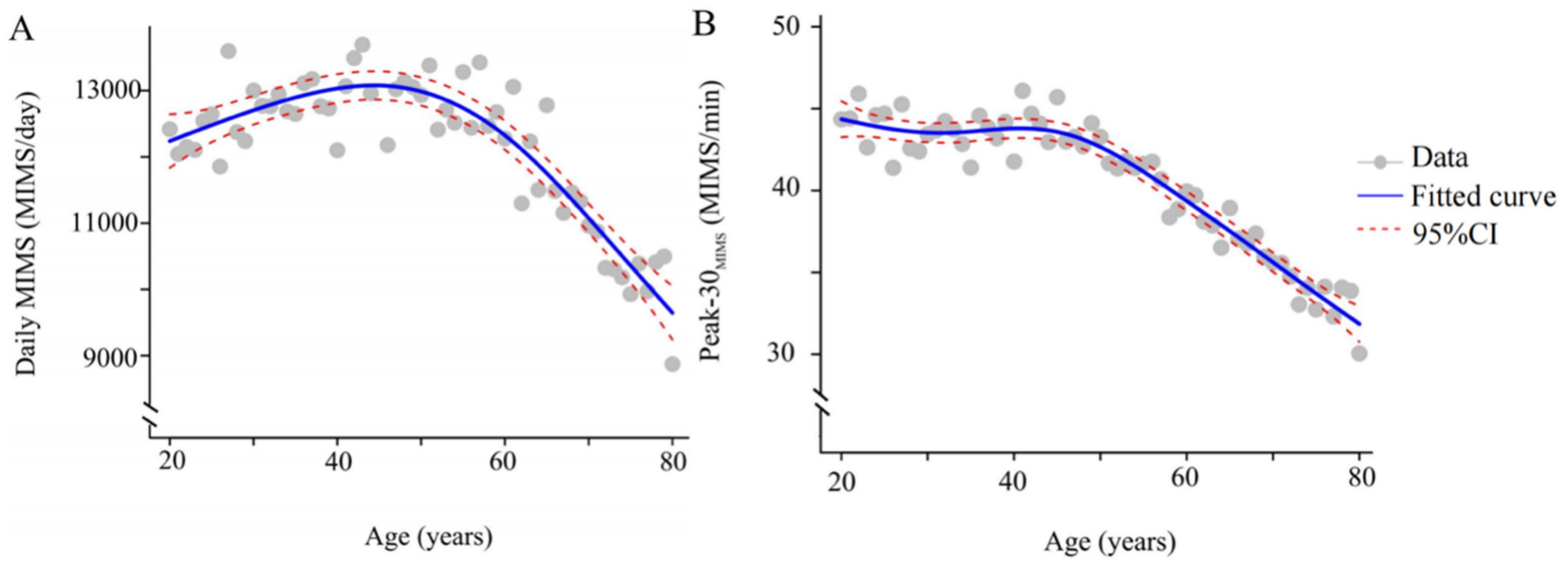
Smoothing spline plots of daily MIMS **(A)**, Peak-30_MIMS_ **(B)** and age. Blue solid lines represent the fitted curve between age and MIMS metric. Red dotted lines represent the 95% CI. Data represent the weighted mean MIMS metrics. MIMS, monitor-independent movement summary; Daily MIMS, daily accumulated MIMS; Peak-30_MIMS_, peak 30-min MIMS.

The study included 8,597 participants (weighted population: 184,156,375, age = 49.26 ± 17.56 years, 4,417 (52%) females), and the characteristics of participants with depression symptoms are detailed in Table 1. Participants without depression symptoms demonstrated greater volume and intensity of PA. The smoothing plots in Figure 2 indicate that Daily MIMS initially rises and then falls with increasing age, in contrast to Peak-30 _MIMS_, which monotonically decreases with age.

**Table 1:** The characteristics by depression symptoms from NHANES 2011 to 2014.

	All	Non-depression symptoms	With depression symptoms	*P*
	*N* = 8,597	*N* = 7,793	*N* = 804	
Daily MIMS	12,265 ± 4,373	12,335 ± 4,377	11,581 ± 4,271	<0.01
Peak-30_MIMS_	40.3 ± 13.8	40.6 ± 14.2	37.7 ± 9.4	<0.01
Sex				<0.01
Male	4,180 (48.0%)	3,904 (50.1%)	276 (34.3%)	
Female	4,417 (52.0%)	3,889 (49.9%)	528 (65.7%)	
Race/ethnicity				<0.01
Mexican American	986 (8.2%)	901 (11.6%)	85 (10.6%)	
Other Hispanic	809 (5.7%)	693 (8.9%)	116 (14.4%)	
Non-Hispanic White	3,581 (67.8%)	3,237 (41.5%)	344 (42.8%)	
Non-Hispanic Black	2004 (11.0%)	1816 (23.3%)	188 (23.4%)	
Non-Hispanic Asian	954 (4.5%)	924 (11.9%)	30 (3.7%)	
Other Race	263 (2.8%)	222 (2.9%)	41 (5.1%)	
Education				<0.01
<High school	644 (4.3%)	545 (7.0%)	99 (12.3%)	
Completed high school	3,045 (31.9%)	2,685 (34.5%)	360 (44.8%)	
>High school	4,906 (63.8%)	4,561 (58.5%)	345 (42.9%)	
BMI				<0.01
Low weight	136 (1.4%)	122 (1.6%)	14 (1.8%)	
Normal weight	2,385 (27.2%)	2,217 (28.7%)	168 (21.2%)	
Overweight	2,735 (33.5%)	2,534 (32.8%)	201 (25.3%)	
Obesity	3,264 (37.9%)	2,854 (36.9%)	410 (51.7%)	
Hypertension				<0.01
No	5,101 (62.9%)	4,723 (60.6%)	378 (47.0%)	
Yes	3,496 (37.1%)	3,070 (39.4%)	426 (53.0%)	

MIMS, monitor-independent movement summary; Daily MIMS, daily accumulated MIMS; Peak-30_MIMS_, peak 30-min MIMS; BMI, body mass index. Low weight: <18.5 kg/m^2^, normal weight: 18.5–24.9 kg/m^2^, overweight: 25–29.9 kg/m^2^, obesity ≥30 kg/m^2^.

### 3.2 Association between weighted quartiles of MIMS metrics and depression symptoms

The Daily MIMS values at the 25th, 50th, and 75th percentiles were 9855.92, 12461.04, and 15071.64, respectively, and for Peak-30_MIMS_, these values were 35.49, 40.67, and 46.25, respectively, with all data divided into quartiles (Q1-Q4) according to weighted percentiles. Table 2 illustrates the association between the Daily MIMS, Peak-30_MIMS_ (classified into quartiles based on weighted percentiles) and depression symptoms, after adjusting for all covariates. Increased MIMS metrics was associated with lower depression symptoms risk. Specifically, participants in the highest quartile (Q4) for Daily MIMS and Peak-30_MIMS_ had a 39% [Odds ratio (OR) = 0.61, 95% confidence interval (95% CI): 0.45–0.82] and 54% (OR = 0.46, 95% CI: 0.34–0.63) lower risk of depression symptoms compared to those in the lowest quartile (Q1).

**Table 2:** Relationship between physical activity separated by weighted quartiles and depression symptoms among adults.

	Daily MIMS		Peak-30_MIMS_	
	OR (95% CI)	*P*	OR (95% CI)	*P*
Q1	1 (Ref)		1 (Ref)	
Q2	0.84 (0.65, 1.08)	0.16	0.85 (0.65, 1.11)	0.21
Q3	0.67 (0.53, 0.86)	<0.01	0.74 (0.57, 0.96)	0.03
Q4	0.61 (0.45, 0.82)	<0.01	0.46 (0.34, 0.63)	<0.01

Q1-Q4 represents weighted quartiles of Daily MIMS (Q1: <9855.92, Q2: 9855.92 – < 12461.04, Q3: 12461.04 – < 15071.64, Q4: ≥15071.64) and Peak-30_MIMS_ (Q1: <35.49, Q2: 35.49 – < 40.67, Q3: 40.67 – < 46.25, Q4: ≥46.25). MIMS, monitor-independent movement summary; Daily MIMS, daily accumulated MIMS; Peak-30_MIMS_, peak 30-min MIMS; OR, Odds ratio; 95% CI, 95% confidence interval.

### 3.3 Dose–response associations between MIMS metrics and depression symptoms

After adjusting for covariates, a significant dose–response association was exhibited between MIMS metrics and the risk of depression symptoms in models with either Daily MIMS or Peak-30_MIMS_ alone, as shown in Table 3. Specifically, every additional 1,000 MIMS/day in Daily MIMS and 1 MIMS/min in Peak-30_MIMS_ were associated with a 5% (OR = 0.95, 95% CI: 0.94, 0.98) and 2% (OR = 0.98, 95% CI: 0.97, 0.99) reduction in the risk of depression symptoms, respectively. The association between Peak-30_MIMS_ (OR = 0.98, 95% CI: 0.97, 0.99) and depression symptoms remained statistically significant in models with both Daily MIMS and Peak-30_MIMS_ included, while the significance of Daily MIMS was not maintained (*p* = 0.60).

**Table 3:** Dose–response relationship between physical activity and depression symptoms among adults.

	Daily MIMS (1,000 MIMS/day)		Peak-30_MIMS_ (MIMS/min)	
	OR (95% CI)	*P*	OR (95% CI)	*P*

Model 1	0.95 (0.94, 0.98)	<0.01	–	–
Model 2	–	–	0.98 (0.97, 0.99)	<0.01
Model 3	0.99 (0.95, 1.03)	0.60	0.98 (0.97, 0.99)	0.02

Model 1 only included Daily MIMS and all covariates. Model 2 only included Peak-30_MIMS_ and all covariates. Model 3 included both Daily MIMS and Peak-30_MIMS_, and all covariates. MIMS: monitor-independent movement summary. Daily MIMS, daily accumulated MIMS; Peak-30_MIMS_, peak 30-min MIMS; OR, Odds ratio; 95% CI, 95% confidence interval.

### 3.4 Analysis of restricted cubic spline

**Figure 3:**
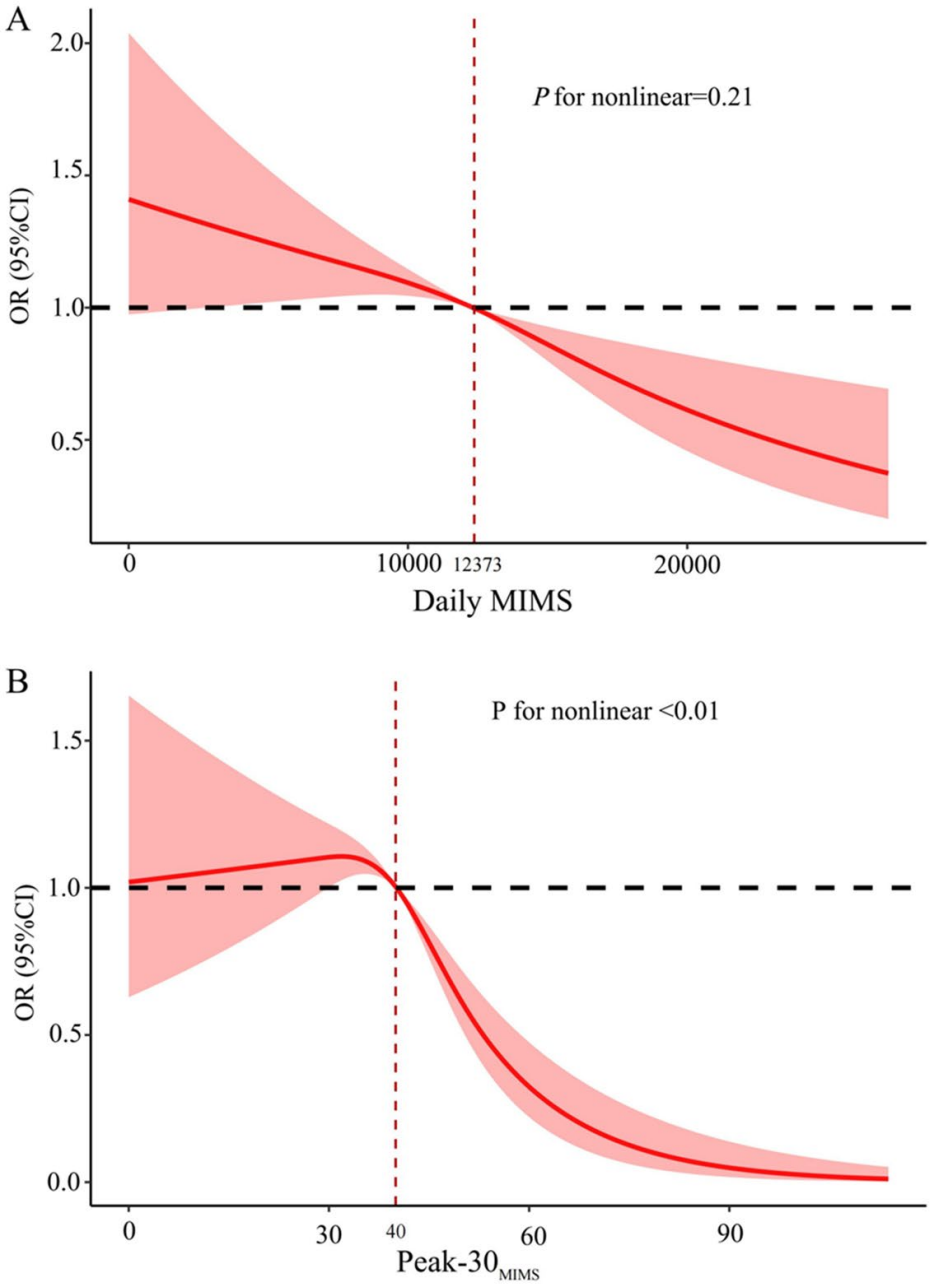
Dose–response associations between daily MIMS **(A)** and Peak-30_MIMS_ **(B)** with depression symptoms. MIMS, monitor-independent movement summary; Daily MIMS, daily accumulated MIMS; Peak-30_MIMS_, peak 30-min MIMS; OR, Odds ratio; 95% CI, 95% confidence interval.

After adjusting for covariates in the restricted cubic spline analysis depicted in Figure 3, a significant nonlinear relationship was identified between Peak-30_MIMS_ and the risk of depression symptoms (*P* for nonlinear < 0.01). Unlike Daily MIMS, which did not exhibit such a relationship (*P* for nonlinear = 0.21). The risk of depression symptoms decreases with higher PA volume, and the risk of depression symptoms falling below a relative risk of 1 when the Daily MIMS reaches approximately 12,373 MIMS/day. Initially, the risk of depression symptoms rises with increasing PA intensity before declining, and it falls below a relative risk of 1 at about 40 MIMS/min of Peak-30_MIMS_.

## 4 Discussion

To our knowledge, this is the first study to investigate the dose–response relationship between PA volume, intensity and depression symptoms using nationally representative population of US adults who worn wrist physical activity monitor. Our results indicated that higher Daily MIMS and Peak-30_MIMS_ were associated with lower depression symptoms risk. However, the association between Daily MIMS and depression symptoms became nonsignificant when both Daily MIMS and Peak-30_MIMS_ were included in the analysis. In other words, among adults with similar PA volumes, those who participated in higher intensity activities during the day had a decreased risk of depression symptoms, highlighting the significant link between peak effort (intensity) and depression symptoms.

Previous studies have found that an increase in PA volume, as measured by the global physical activity questionnaire, is associated with a reduction in depression (27, 29). For example, He et al. (27) used NHANES 2007–2018 data and found that leisure-time PA was associated with a lower risk of depressive symptoms at any amount. A systematic review and meta-analysis encompassing over 2 million person-years from 15 prospective studies found that individuals accumulating at least half the recommended physical activity volume (4.4 marginal metabolic equivalent task hours per week, 4.4 mMET-h/week) had an 18% (95% CI: 13–23%) lower risk of depression, while those reaching 8.8 mMET-h/week had a 25% (95% CI: 18–32%) reduction in risk, compared with adults who not reporting any activity (30). Our findings are consistent with these previous results, showing that PA volume measured by Daily MIMS using accelerometry is inversely associated with the risk of depression symptoms. However, the significance of PA volume in relation to depression symptoms were negated when controlling for PA intensity. The main reason for this observation is largely attributed to the methodology of PA measurement (31, 32). Specifically, there was independence between PA volume and intensity measured by accelerometry. In contrast, the calculation of PA intensity derived from questionnaires often involved the PA volume (33, 34). Hence, this study examined a more extensive range of PA, shedding additional light on the association between PA and depression symptoms.

Numerous studies have demonstrated that PA volume is negatively associated with depression (27, 29), but the benefits of depression prevention may vary with the intensity of PA (35, 36). In a randomized controlled trial during COVID-19 confinement, high intensity interval training demonstrated greater effectiveness in reducing depression symptoms compared to moderate-intensity training (37). Huang et al. (36) found that increased vigorous physical activity (VPA) was associated with lower depressive symptoms in US adults, with each additional hour or day per week of VPA reducing depression risk by 6 and 12%, respectively. Yang et al. (35) found that a higher proportion of VPA to MVPA might be correlated with a lower risk for depression. However, these studies assessed PA levels using the GPAQ. In contrast, our study used accelerometry data and found that each additional 1 unit increase in Peak-30_MIMS_ was associated with a 2% reduction in depression risk, and identified a non-linear relationship between PA intensity and the risk of depression symptoms, as Peak-30_MIMS_ increases, there is no significant change in the risk of depression symptoms. However, once Peak-30_MIMS_ exceeds approximately 40 MIMS/min, a notable decrease in depression risk is observed. This threshold effect suggests that while moderate increases in PA intensity may not immediately impact depression symptoms, surpassing a certain intensity threshold can lead to significant mental health benefits. This finding underscores the importance of not only encouraging PA, but also considering the intensity at which individuals engage in these activities to maximize their potential antidepressant effects. Even after adjusting for Daily MIMS, those who participated in higher intensity activities during the day had a decreased risk of depression symptoms, indicated that PA intensity is a main driver in lowering the risk of depression symptoms.

The underlying mechanisms linking PA intensity to depression symptoms remain largely unexplored. From the physiological aspects, inflammation hypothesis has been proposed (38). Depression-related inflammatory cytokines encompass tumor necrosis factor alpha (TNF-*α*), interleukin-1 beta (IL-1β) (39–41), and interleukin-6 (IL-6), which is secreted by skeletal muscle (42). Elevated TNF-*α*levels are potentially implicated in the etiology of depression (43, 44). Lowered IL-1β levels are correlated with a decrease in depressive symptoms in individuals with major depressive disorder (44). Several studies have shown that IL-6 levels rise following high-intensity PA (42, 45) and such acute elevations in IL-6 can inhibit TNF-*α* production (46, 47). A randomized controlled trial revealed that 6 weeks of high-intensity interval training can decrease levels of TNF-α and IL-1β (48).

Moreover, PA acts as a stressor that triggers neural and physiological adaptations specific to the type (49, 50), intensity (35, 36), and duration of PA (36). These adaptations include changes in endorphin levels (51), reduction of pro-inflammatory cytokines (39–41), and enhanced neuroplasticity (52), particularly through the modulation of neurotrophic factors such as brain-derived neurotrophic factor (BDNF) (53). For instance, BDNF has been shown to play a crucial role in promoting synaptic plasticity and neurogenesis, which are essential for mood regulation and cognitive function (53). Higher levels of PA can enhance cerebral blood flow, promote the proliferation of neurotrophic factors such as BDNF, and augment synaptic plasticity, thereby improving neural efficiency (53).

Psychological mechanisms involved in the relationship between PA intensity and depression prevention include self-efficacy theory (54) and the health belief model (55). A previous study has shown that the relationship between PA intensity and the prevention of depression in Chinese individuals is moderated by perceived susceptibility and self-efficacy (56). Consequently, while fatigue may follow high-intensity physical activity, there can be enhancements in an individual’s self-efficacy and self-perception, which in turn may lower the severity of depressive symptoms.

Despite our adjustment for demographic factors, such as sex, race/ethnicity, education, and age, the potential for residual confounding remains. It is crucial to recognize additional factors that could influence both PA levels and depressive symptoms. For instance, chronic illnesses (28) and disabilities (57, 58) can significantly affect an individual’s ability to engage in PA and concurrently elevate the risk of depression. Socioeconomic stressors, such as financial instability, and the use of green space have also been linked to depression risk (59). Nutritional status is another significant factor, a study by Huang et al. (60) using machine learning revealed that specific nutrients, including potassium and vitamin E, along with the diversity of food and beverage intake, are significantly related to depression risk. Sleep duration is independently associated with depression, with a U-shaped relationship indicating that both insufficient and excessive sleep can increase depression risk (61). Future research should conduct sensitivity analyses to further explore how these confounding factors might affect the observed associations. Future research should conduct sensitivity analyses to further explore how these confounding factors might affect the observed associations.

The use of MIMS-based accelerometry offers significant practical implications for public health interventions and clinical screening tools. MIMS metrics provide an objective and comprehensive assessment of PA, which can be particularly useful in large-scale public health initiatives. For example, incorporating MIMS data into community-based programs can help tailor interventions to individuals with varying PA levels and intensities, thereby enhancing the effectiveness of depression prevention strategies. In clinical settings, MIMS-based accelerometry can serve as a valuable screening tool to identify patients at risk for depression, allowing for early intervention and personalized treatment plans. Future research should explore the feasibility and effectiveness of integrating MIMS metrics into existing public health and clinical frameworks to improve mental health outcomes.

This study has several strengths. Accelerometers were utilized for a comprehensive evaluation of PA, replacing self-reported questionnaires. Moreover, the peak index was introduced to represent the PA intensity. Finally, our results represent the general US adult’s population, enhancing the generalizability of the study. This study also has several limitations. Only cross-sectional data was employed for the estimations in this study, and the causal relationship between PA and depression cannot be inferred. Although relevant variables were considered, the removal of confounding factors presents a considerable challenge. Future studies that are more relevant are anticipated to confirm our results and are encouraged to utilize the MIMS algorithm for analyzing wrist-worn physical activity monitor data.

## 5 Conclusion

Higher levels of PA, including both volume and intensity, correlate with lower risk of depression symptoms. There are no significant associations between PA volume and depression symptoms after adjusting for PA intensity. Our findings suggest that promoting higher-intensity physical activity may be a valuable strategy for depression prevention and management.

## Data Availability

The datasets presented in this study can be found in online repositories. The names of the repository/repositories and accession number(s) can be found at: https://www.cdc.gov/nchs/nhanes/.
